# Preparation, characterization, and binding profile of imprinted semi-IPN cryogel composite for aluminum

**DOI:** 10.3906/kim-2002-36

**Published:** 2020-08-18

**Authors:** Shahnila SHAH, Huma SHAİKH, Najma MEMON, Muhammad Iqbal BHANGER, Tahira QURESHİ, Humaira KHAN, Adil DENİZLİ

**Affiliations:** 1 National Center of Excellence in Analytical Chemistry, University of Sindh, Jamshoro Pakistan; 2 H. E. J. R.I.C., I.C.C.B.S. University of Karachi,Karachi, Sindh Pakistan; 3 Department of Chemistry, Biochemistry Division, Hacettepe University, Ankara Turkey; 4 Dr. M.A. Kazi institute of Chemistry, University of Sindh, Jamshoro Pakistan

**Keywords:** Semiinterpenetrating polymeric networks, imprinted polymer, cryogel, composites, aluminum, water purification

## Abstract

Human body is greatly exposed to aluminum due to its high abundance in the environment. This nonessential metal is a threat to the patients of chronic renal disorders, as it is easily retained in their plasma and quickly accumulates in different tissues. Thus, there is great need to remove it from the aqueous environment. In this study, Al^3+^ imprinted semiinterpenetrating polymer network (semi-IPN)-based cryogel composite was prepared and applied for the purification of environmental and drinking water samples from aluminum. Poly (2-hydroxyethyl methacrylate) (pHEMA) discs were produced via cryogenic treatment and imprinted semi-IPN was introduced to the 3-(trimethoxysilyl) propyl acrylatemodified macroporous cryogel discs. The adsorption properties and selectivity of the aluminum (III) imprinted semi-IPN cryogel composite were studied in detail. The imprinted semi-IPN cryogel composite showed good selectivity towards aluminum (III) ions with the imprinting factor (IF) of 76.4 in the presence of competing copper (II), nickle (II), and iron (III) ions. The maximum adsorption capacity of 271 μmol g^-1^ was obtained for aluminum (III) at pH 7.0 within 10 min using imprinted semi-IPN cryogel composite. The good selectivity and reusability of aluminum (III)-imprinted semi-IPN cryogel composite makes this material an eligible candidate for the purification of drinking water from aluminum (III) leaving important minerals remained in the water.

## 1. Introduction

Increasing demand to obtain clean and safe aqueous environment has boosted up the progress of green synthetic materials that can clean the water systems efficiently. In this regard, consideration is given to the synthesis of novel macroporous and water-compatible polymers. Macroporous polymeric materials have already found their immense applications in different fields including biology, chemistry, bioengineering, and biotechnology [1]. These synthetic materials can be used for the quick isolation and purification of a range of ions and molecules [2]. Among the water-compatible macroporous polymers; macroporous hydrogels are the most popular due to their unique heterogeneous open porous structure which significantly increases their equilibrium sorption properties and allows unhindered diffusion of solutes, nano- and even microparticles [3]. Macroporous hydrogels are a class of hydrogels with porosities and/or feature sizes in the micron range that can effectively combine the desirable properties of conventional hydrogels with the transport properties of conventional porous media. The wide range of preparation techniques, allows for precise control over pore size, pore orientation, and gel morphology, leading to highly functional materials for targeted applications including filtration, directing cell growth, and templating other materials [4]. Although increased hydrogel porosity is extremely beneficial for rapid swelling and diffusion of solutes throughout the network, most preparation techniques exhibit limitations. First, the typically desirable increased pore volume is directly associated with a decrease in overall mechanical integrity of the network, as a fraction of the gel phase is replaced by water. Given that hydrogels are already relatively weak materials, additional mechanical losses significantly limit the end use of many macroporous hydrogels both in terms of biological applications as well as separation-based applications [5].

Cryogels are another unique class of porous hydrogels with neutral, anionic, and cationic functionalities. Cryogels are categorized as innovative class of adsorbents because of their exceptional structural properties and flow dynamics [6]. They are highly cross-linked 3D-materials having a huge arrangement of interconnected pores with sizes ranging from 1 to 100 μm [7].

Cryogels with the macropores can be incorporated via free radical polymerization of monomer solutions using cryogenic treatment [8]. During the cryogenic treatment, solvent molecules act as porogens to craft interrelated flow channels which allow high flow rate of liquid samples during cryogel’s adsorption applications and overcome all kinds of plugging and diffusion problems encountered during purification process [9]. In addition, a highly cross-linked polymeric network can be obtained by increasing the cryo-concentration of monomers in unfrozen liquid microphase. Water is the most commonly used porogen to synthesize cryogels using water-soluble monomers. In addition to excellent flow-dynamics and macro porosity, cryogels exhibit high physical, chemical, and mechanical stability which can be modified by choosing appropriate water-soluble monomers [10]. Thus, cryogels are promising materials for a vast range of applications in the numerous research fields including food safety, water treatment, environmental sciences, chemistry, and biotechnology [11].

Another major challenge for the adsorbents used in separation and removal applications is their selectivity or recognition for the target molecule/ion in a vast collection of similar molecules/ions. Lately, imprinted materials have captivated substantial attention as they offer high selectivity towards particular target, mechanical stability, easy synthesis, and low cost [12]. Molecular imprinting is a technique of grafting the recognition sites in a macromolecular matrix by introducing template ion/molecule during the synthesis. These sites memorize the shape and size of template and get activated to recognize and detect it whenever it comes into contact with them again [13]. Molecular imprinting has been used successfully for imprinting of small molecules, large molecules and metal ions [14].

Imprinted cryogels are an excellent substitute to nonselective adsorbents with a number of benefits including short diffusion path, very short residence time, large pores and a low pressure drop [15]. However, due to the presence of sizeable pores inside the cryogel, they offer poor adsorption capacity for selected molecules or ions [16]. In actual adsorption phenomenon, it is very important to enhance the binding capability of highly macroporous cryogels. Hence, composite cryogels would be a great option for enhancing the surface area [17]. This approach results in the production of cryogels with compact selection strategy and improved adsorption properties. Variety of methods have been reported to prepare composite cryogels, they include embedding of microbeads [18], embedding of polymer particles [19], embedding of nanoparticles [20], and interlocking of interpenetrating polymer networks (IPN) [21] into the cryogels.

 Interpenetrating polymer networks (IPN) are a blend of two or more cross-linked polymers, which are synthesized in such a way that one of the polymer networks is interpenetrated into the other during the synthesis [22]. In last few decades, IPNs have appeared as one of the most competent polymeric materials and have found applications in toughened plastics, coatings, damping materials, functional materials, and reinforced rubbers because of their exceptional strength and compatibility [23]. Additionally, IPN technology is an outstanding method for stable integration of two polymer networks with different properties or different functions by physical entanglements [24].

IPNs enhance the mechanical strength, biodegradability, stability, and solutes diffusion ability of sorbent. They have been considered the best interlocked structures in the cross-linked networks [25]. In IPNs one polymer chain permeates into another polymeric network at molecular level with or without chemical bonds [26].Chemical crosslinking between these polymers leads to improved mechanical properties and thermal stability [27]. IPNs have been widely used in the last decade to generate more sophisticated and more efficient hydrogels. Some physical properties of hydrogels may be improved by preparing semiinterpenetrating polymer networks (semi-IPNs). Water sorption property of hydrogels or semiinterpenetrating polymer networks accounts for a great number of biomedical and technological applications such as drug delivery systems, artificial implants, contact lens, enzyme immobilization, catheters, wound dressings, biosensors, superabsorbents [28]. The semi-IPN composite cryogels were reported by Dragan et al. [29] for the improved sorption of methylene blue. Cryogels have already been proved to be better hydrogels in terms of mechanical strength, pore size, reusability, and water uptake [30]. Thus, semi-IPN cryogel composites can be very effective adsorbents for the purification of aqueous environment [31].

In this study, we report aluminum-imprinted semi-IPN cryogel composites for selective removal of aluminum from aqueous systems. The imprinted semi-IPN cryogel composites were successfully employed for enhanced and efficient removal of Al^3+^from aqueous systems; precisely river water and drinking water. The synthesized imprinted semi-IPN cryogel composites were characterized thoroughly and examined for their kinetics, adsorption capacity, selectivity, and reusability for aluminum removal.

## 2. Experimental

### 2.1. Reagents and chemicals

Each reagent used in this study was of analytical grade and solution preparation was done in deionized water. Each working solution was prepared from its stock solution by diluting it in an appropriate volume. 2-Hydroxyethyl methacrylate (HEMA) was purchased from Alfa Aesar, England. N, N’-Methylenebis bis acrylamide (MBAAm) was obtained from ICN, Germany. Ammonium persulfate (APS) was supplied by Duksan, Korea. 3-(Trimethoxysilyl)propyl methacrylate 98%, 2, 2’-Azobis(2-amidinopropane) dihydrochloride, and ethylenediaminetetraacetic acid (EDTA) were purchased from Sigma Aldrich, USA. Chromotrop 2 R was provided by Fluka, Chemika, Switzerland. Other all HPLC grade solvents and analytical grade reagents were provided by Merck AG, Darmstadt, Germany.

### 2.2. Instrumentation

Double beam UV-Visible spectrophotometer, (Agilent-Carry 100, England) equipped with the quartz cells of 1.0 cm path length had been used for the detection of Al^3+^. The details of spectrophotometric determination of aluminum are given in supplementary data under section S1.

### 2.3. Preparation of Al^3+^imprinted semi-IPN cryogel composite

The synthesis of pHEMA cryogel was carried out using the procedure reported elsewhere with minor modification [32]. Precisely, 0.3 g of MBAAm and 1.3 mL of HEMA were dissolved in 10 mL of DI water and reaction mixture was kept in an ice bath for 10 min. APS and TEMED were then added to the reaction mixture in order to initiate the free radical polymerization for the preparation of cryogel. Next, 20 mg of APS was added into the reaction mixture and cooled for 2–3 min in an ice bath, 85 μL of TEMED was then added, and the solution was stirred for 1 min. Both the APS and TEMED were taken as 1% (w/v) of the total monomer. Subsequently, the monomers solution was poured in between two glass plates, which were separated by a spacer having thickness of 1.5 mm. Free radical crosslinking copolymerization of HEMA with MBAAm was conducted at –16 °C for 24 h. After thawing, the cryogel was cut into small discs. The next step was surface modification via silanization. The surface of the discs was modified by soaking them in the 50% solution of 3-(Trimethoxysilyl)propyl methacrylate prepared in methanol and water (1:1) for 48 h. After thorough washing and drying, the discs were immersed in 0.05 M, 2, 2’-Azobis(2-amidinopropane) dihydrochloride (initiator) for 3 h. The template-monomer complex was prepared by adding 0.012 mol of HEMA (1.5mL) to the 10 mL aqueous solution containing 0.012 mol of aluminum (2.62 g of aluminum nitrate). This prepolymerization complex of Al^3+^ and HEMA was finally allowed to polymerize in the presence of initiator soaked silanized cryogel discs for 18 h at 55 °C. The resulting Al^3+^-imprinted semi-IPN cryogel composite discs were washed thoroughly to remove Al^3+^ions.

### 2.4. Characterization of Al^3+^imprinted semi-IPN cryogel composite

The Al^3+^ imprinted semi-IPN cryogel composite was thoroughly characterized using scanning electron microscope, energy dispersive spectroscopy, Fourier-transform infrared spectroscopy, and swelling studies. The details are given in supplementary data under Section S2.

### 2.5. Batch sorption experiments

To assist the best pH for maximum binding, 10-mL solutions containing 370 μM of Al^3+^ ions were prepared. The pH of solutions was maintained using phosphate buffers in the range of 3.0 to 9.0. Each solution containing 1 disc of Al^3+^-imprinted semi-IPN cryogel composite (55 mg) was mechanically shacked for 2 h at 50 rpm. Subsequently, discs were removed and the unadsorbed amount of Al^3+^was estimated by using UV-visible spectrophotometer as explained in Section S1. The pH study of nonimprinted semi-IPN cryogel composite was also performed in a similar way. The readings were taken three times and were represented as the mean. To estimate the binding kinetics of synthesized imprinted semi-IPN cryogel composites, nine different flasks of 25 mL were taken, each containing 10 mL of 370 μM aqueous solution of Al^3+^ and one Al^3+^-imprinted cryogel composite disc (55 mg). The flasks were mechanically shacked at 50 rpm for 5, 10, 15, 20, 25, 30, 45, 60, and 120 min at ambient temperature. Subsequently, discs were removed from the mixture at particular recorded time and solutions were analyzed for their aluminum content as given in Section S1. The same method was used to evaluate the binding kinetics of nonimprinted semi-IPN cryogel composite. The data for each sample were taken three times and presented as the mean.

Evaluation of adsorption capacity of Al^3+^-imprinted and nonimprinted semi-IPN cryogel composites for Al^3+^ ions was performed via batch adsorption experiments. Precisely, 10 mL of aqueous solutions containing different concentrations of Al^3+^ ions ranging from 35 to 5550 μM, maintained at pH 7.0 were treated with 55 mg (dry weight of 1 cryogel disc) of the Al^3+^ -imprinted and nonimprinted semi-IPN cryogel composites for 10 min at room temperature with continuous shaking (50 rpm). After 10 min, the discs were removed and the remaining mixture was analyzed for its aluminum content as given in Section S1. The data were recorded three times and represented as the mean.

### 2.6. Selectivity experiments

Al^3+^-imprinted semi-IPN cryogel composite was investigated for its selectivity towards aluminum by adsorbing Al^3+^, Ni^2+^, Fe^3+^, and Cu^2+^ ions from their aqueous solutions using batch adsorption experiments. The salts used as the source Ni^2+^, Fe^3+^, and Cu^2+^ ions were Ni(NO_3_)_2_, FeCl_3_. 6H_2_O, and CuSO_4_. 5H_2_O, respectively. A solution (10 mL) containing 10 μg mL−1 of each metal was treated with Al^3+^-imprinted and nonimprinted semi-IPN cryogel composite disc at pH 7.0 with continuous shaking for 10 min. After reaching sorption equilibrium, the remaining concentration of metal ions (total Al^3+^, Ni^2+^, Fe^3+^, and Cu^2+^) in their aqueous solutions was estimated as described in Section S1. Each experiment was repeated three times and represented as the mean.

### 2.7. Removal studies on real aqueous samples

The removal efficiency of Al^3+^-imprinted semi-IPN cryogel composite was also evaluated for real environmental samples such as river and tap water. Six batch adsorption experiments were performed on different volumes (50, 100, and 150 mL) of river water and tap water samples spiked with 2.0 μM of Al^3+^. The mixtures were shaken to allow the adsorption of Al^3+^ ions onto the Al^3+^-imprinted and nonimprinted semi-IPN cryogel composite disc for 10 min at 50 rpm. All solutions were filtered after removing discs and analyzed using the procedure given in section S1 of the supplementary data. The data was represented as the mean of each experiment repeated three times.

### 2.8. Desorption and reuse

The reusability of Al^3+^-imprinted and nonimprinted semi-IPN cryogel composites was checked 10 times after its regeneration. Discs were washed collectively after every experiment. Thorough washing of imprinted and nonimprinted semi-IPN cryogel composites for the removal of aluminum was preceded by sequential washing steps. Precisely, the discs were first washed with the 6:4 ratio of methanol and water for 24 h and then with 2.5 mM EDTA for 48 h. Finally, cryogel discs were treated with 0.1 M HNO_3_ for 3 h and thoroughly washed with DI water to remove all the HNO_3_. Optimal template removal was marked by no further change in the quantity of Al^3+^ ions detected in the supernatant liquid under UV-Vis spectrophotometer.

## 3. Results and discussions

### 3.1. Preparation of Al^3+^-imprinted semi-IPN cryogel composite discs

**Figure 1:**
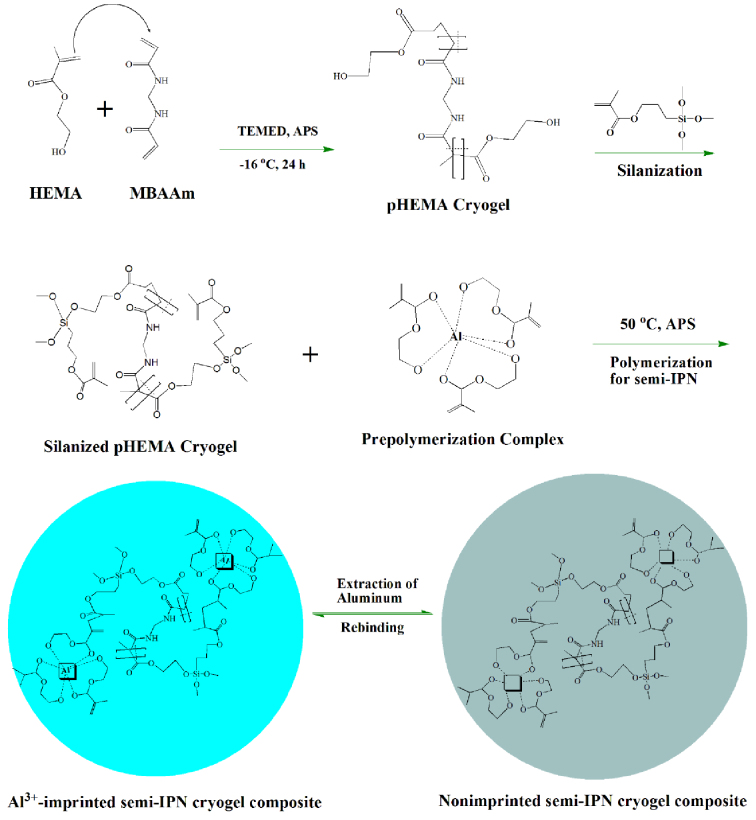
Schematic representation of synthesis of Al^3+^-imprinted semi-IPN cryogel composite.

As shown in Figure 1, synthesis of Al^3+^-imprinted semi-IPN cryogel composite was a multistep process, which involves synthesis of pHEMA cryogel discs, modification of synthesized cryogel discs with 3-(Trimethoxysilyl)propyl methacrylate, and finally the preparation of imprinted semi-IPN in the presence of modified pHEMA cryogel discs. Different studies reported removal of aluminum with microspheres of poly EGDMA-HEMA incorporated with different dyes, i.e. Congo Red, Alkali Blue 6B, and Cibacron Blue F3GA [33]. In another report, magnetic beads of poly(2-hydroxyethyl methacrylate) attached with alizarin have been used for aluminum removal from both, dialysate fluid and drinking water [34]. However, the use of dyes as recognition moiety does not prove to be significant due toxicity concern as there are chances of leaching of the dye into the aqueous environment. Moreover, the adsorption–desorption process is rather time-consuming. The chelating agents like Sodium di-(n-octyl) phosphinate have also been used to remove aluminum from aqueous environment [35]. The removal is effective at varying pH values that result in competition between Al^3+^ and Ca^2+^ for ligand, as the ligand gets free after the formation of an insoluble aluminum hydroxide. Moreover, the high adsorption capacity values could only be achieved at acidic condition which is not suitable for drinking water. Removal of aluminum from aqueous bodies has also been examined by using BDH activated carbon and date-pit activated carbon [36], though this can work well in slightly acidic conditions. However, newly synthesized Al^3+^-imprinted semi-IPN cryogel composite gives maximum adsorption at neutral condition. Moreover, it is effective for adsorbing aluminum due to its high adsorption capacity.

Thus, in the present work motivation for the synthesis of pHEMA cryogel discs was the hydrophilic nature of pHEMA; as the synthesized imprinted semi-IPN composite cryogel was applied on aqueous system for removal of aluminum. Thus, its water and environmental compatibility was kept in mind during synthesis process. After synthesizing pHEMA cryogel discs via cryogelation, the discs were silanized using 3-(Trimethoxysilyl)propyl methacrylate. The imprinting of Al^3+^ was performed in semi-IPN using HEMA as functional monomer. The synthesis of semi-IPN of HEMA was accomplished by soaking the silanized cryogel discs in Azobis(2-amidinopropane) dihydrochloride (initiator). This is a relatively simple and more convenient method for the synthesis of semi-IPNs as it leads to fiber-like semi-IPNs evenly distributed into the macroporous structure of pHEMA cryogel discs. Moreover, to the best of our knowledge, imprinting of aluminum ions has not been yet introduced to interpenetrating polymer networks. The purpose of choosing 3-(Trimethoxysilyl)propyl methacrylate for silanization was to introduce sufficient acrylate groups into the pHEMA cryogel that may assist the grafting of interpenetrating polymer network and produce semi-IPN. The synthesized imprinted semi-IPN cryogel composite was found to be efficient for the removal of aluminum from aqueous systems. The imprinted semi-IPN cryogel composite showed good kinetics, specificity, and capacity for the adsorption of aluminum. Moreover, it was robust and reusable.

### 3.2. Characterization

#### 3.2.1. Scanning electron microscopy (SEM) and energy dispersive spectroscopy (EDS)

**Figure 2:**
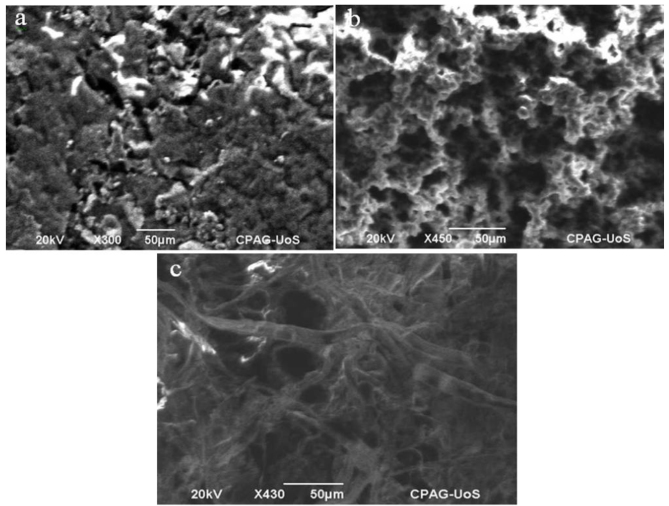
SEM images of (a) pHEMA cryogel, (b) Silanized pHEMA cryogel, and (c) Al^3+^-imprinted semi-IPN cryogel composite.

The morphology of pHEMA cryogel, silanized pHEMA cryogel, and imprinted semi-IPN cryogel composites was thoroughly studied using SEM. It can be seen from Figure 2a that the morphology of pHEMA cryogel is very smooth. Moreover, pHEMA cryogel is less porous as compared to silanized pHEMA cryogel (Figure 2b). The walls of silanized pHEMA cryogel are more defined and thicker than the walls of pHEMA cryogel due to silanization. However, the structure of Al^3+^-imprinted semi-IPN cryogel composite contains very fine interpenetrating fibers that prove the successful synthesis of Al^3+^-imprinted semi-IPN within the macroporous structure of silanized pHEMA cryogel (Figure 2c). Moreover, the average pore size of pHEMA cryogel was found as 17 μm (STD ±17 μm) with a broad pore size distribution from 3 to 93 μm. The gels showing broader pore size distribution are generally considered weaker and more fragile. However, the average pore size of silanized pHEMA cryogel was found as 9 μm (STD ±5.5 μm) with pore size distribution ranging from 1.6 to 28 μm. The results clearly show that the thickness of walls of silanized pHEMA cryogel is greater than pHEMA cryogel as the size of macropores between the walls has been reduced greatly. Moreover, the pore size distribution has also become narrow as compared to pHEMA cryogel which reflects that silanization process has increased the strength of cryogel [37]. The average pore size of Al^3+^-imprinted semi-IPN cryogel composite was calculated as 7 μm (STD ±4 μm) with a pore size distribution from 1.5 to 26.5 μm. Here, it is noticeable that there is minute change in the pore size of interconnected flow channels within the walls and macropores between the walls of prepared Al^3+^-imprinted semi-IPN cryogel composite as compared to silanized pHEMA cryogel. It may be due to the fact that semi-IPN has been penetrated into the silanized pHEMA cryogel in the form of fibers (Figure 2c).

The EDS analysis of pHEMA cryogel, silanized pHEMA cryogel and Al^3+^-imprinted semi-IPN cryogel composite was also conducted in order to confirm the modifications of pHEMA cryogel. Figure S1a in the supplementary data shows that there are prominent peaks of carbon and oxygen coming from pHEMA cryogel. However, Figure S1b shows prominent peak for “Si” in addition to apparent peaks of carbon and oxygen. This confirms the silanization of pHEMA cryogels. Furthermore, Figure S1c indicates sufficient presence of aluminum in Al^3+^-imprinted semi-IPN cryogel composite in addition with prominent peaks of carbon and oxygen. However, the weight percent of silica has been decreased due to the presence of additional carbon, oxygen, and aluminum from semi-IPN.

#### 3.2.2. Fourier-transform infrared spectroscopy (FTIR)

**Figure 3:**
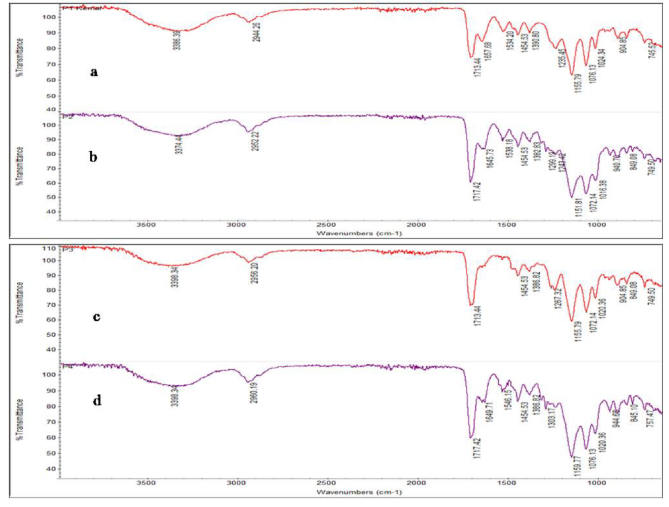
FT-IR spectra of (a) pHEMA cryogel, (b) Silanized pHEMA cryogel, (c) Al^3+^-imprinted semi-IPN cryogel composite, and (d) Nonimprinted semi-IPN cryogel composite.

Synthesized pHEMA, silanized pHEMA, imprinted, and nonimprinted semi-IPN cryogel composites were also characterized through FTIR. Figure 3 reveals FTIR spectra of (a) pHEMA cryogel disc, (b) silanized pHEMA cryogel disc, (c) Al^3+^-imprinted semi-IPN cryogel composite, and (d) nonimprinted semi-IPN cryogel composite. Figures 3a–3d reveal a sharp band due to C=O stretching at 1713, 1717, 1713, and 1717 cm^-1^, respectively, which indicates the presence of monomers in all polymers. It also reveals that C=O stretching is slightly shifting from 1713 cm^-1^ in pHEMA cryogel to 1717 cm^-1^ in silanized pHEMA cryogel due to the presence of 3-(Trimethoxysilyl)propyl methacrylate. Moreover, the intensity of the peak has been increased as well due to the addition of C=O groups from 3-(Trimethoxysilyl)propyl methacrylate. However, this peak shifts to 1713 cm^-1^ again in Al^3+^-imprinted semi-IPN cryogel composite with decreased intensity and remains at 1717 cm^-1^ with approximately the same intensity in nonimprinted semi-IPN cryogel composites. The shift and decrease in intensity of this peak in Al^3+^-imprinted semi-IPN cryogel composite may be due to the interactions of C=O group in pHEMA with aluminum ions. It also proves that in imprinted semi-IPN cryogel composite interactions between template ion and monomers exist. All polymers reveal a prominent peak at 3336 cm^-1^ (pHEMA cryogel), 3374 cm^-1^ (silanized pHEMA cryogel), and 3398 cm^-1^ (Al^3+^-imprinted semi-IPN cryogel composite) due to –OH stretching. These peaks confirm polymerization, and shift in imprinted semi-IPN cryogel composite reveal interaction of –OH groups with template ions as well. C–N stretching can be observed in the region of 1155 cm^-1^ in all polymers. Thus, it is predicted that HEMA is interacting with Al^3+^ due to its carbonyl and hydroxyl groups.

#### 3.2.3. Swelling studies

In order to calculate the volume of total porosity, swelling degree and % swelling, and dry and wet weights of Al^3+^-imprinted and nonimprinted semi-IPN cryogel composites were measured. The total volume of porosity can easily be estimated through uptake of solvent. Since a solvent can only enter into the pores of polymeric networks, Vp (milliliters of pores in 1 g of dry polymer network) can be calculated using the following expression [38]:

(1)mswollen gel-msqueezed gelmswollen gel*100%

Here (msqueezed gel) is the weight of sample obtained after squeezing the free water from the swollen gel matrix.

The swelling ratio and swelling degree for the Al^3+^-imprinted and nonimprinted semi-IPN cryogel composites were calculated by using equations 2 and 3, respectively [32]:

(2)mswollen gel-mdried gelmswollen gel*100%

(3)Sw/w=mwet gel-mdry gelmdry gel

Table 1 shows results of the swelling study. The obtained results revealed that the volume of total macropores is higher in nonimprinted semi-IPN cryogel composites than imprinted, the possible explanation for it is the use of Al(NO_3_)_3_ in imprinted semi-IPN composite cryogel for the imprinting of Al^3+^, which increased the thickness of the walls of cryogel and decreased the total volume of macropores. The addition of Al(NO_3_)_3_ during synthesis process is for accomplishing the imprinting phenomenon. However, Al(NO_3_)_3_ also served here as a salt additive that led to thicker walls of imprinted semi-IPN cryogel composite. This ultimately resulted in higher % swelling and degree of swelling of imprinted semi-IPN cryogel composite.

**Table 1:** Swelling study of synthesized Al^3+^-imprinted and nonimprinted semi-IPN cryogel composites.

	Al^3+^-imprinted semi-IPN cryogel composite	Nonimprinted semi-IPN cryogel composite
V total porosity	20 ±1.5	38 ±1.2
Swelling degree	2.6 ±0.1	2.1 ±0.2
% swelling	72 ±0.8	67.5 ±1.85

### 3.3. Sorption studies

#### 3.3.1. Effect of pH

**Figure 4:**
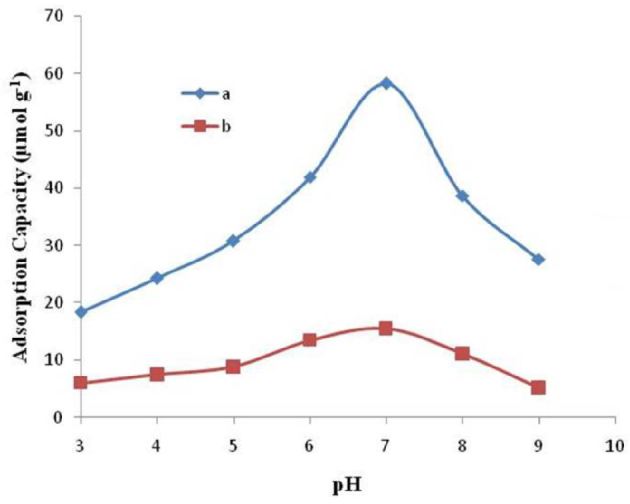
Optimization of pH for maximum adsorption of Al^3+^ on (a) Al^3+^-imprinted semi IPN cryogel composite and (b) Nonimprinted semi-IPN cryogel composite.

Use of particular adsorbents for the removal of metal ion is highly pH-dependent. Ligands are the complexing agents that can satisfy a metal ion’s coordination number to get involved in complex formation. In the absence of these ligands, or at their low concentration, metal ions form hydroxyl complexes after being hydrated with water molecules i.e. Al(OH)^+^_2_, Al(OH)_3_, and Al(OH)^-^_4_. While polynuclear hydroxyl complexes are supposed to be formed at high pH and high concentration of metal ions [39]. As the binding affinity of Al^3+^-imprinted semi-IPN cryogel composite is highly pH-dependent, so the pH is considered an important factor for the adsorption phenomenon [40]. The effect of pH on the adsorption capacity of Al^3+^-imprinted and nonimprinted semi-IPN cryogel composite was determined by the adsorption of Al^3+^ ions (370 μM) at different pH values ranging from 3.0 to 9.0. Figure 4 reveals that there is increase in the binding capacity of Al^3+^-imprinted semi-IPN cryogel composite for Al^3+^ ions up to pH 7.0, and then it starts to decrease with increasing pH for both Al^3+^-imprinted and nonimprinted semi-IPN cryogel composites. The maximum adsorption is found at the pH 7.0. This may be due to the use of HEMA as the functional monomer which is a neutral monomer and binds Al^3+^ effectively at neutral pH. Thus, the synthesized Al^3+^-imprinted semi-IPN cryogel composite is best for the removal Al^3+^ from drinking and surface waters as their pH is usually neutral. Abdullah et al. used Natural zeolite as sorbent for the removal of aluminum ions, but zeolite samples were activated using 1M HCl or NaOH which is not suitable for drinking water [41].

#### 3.3.2. Kinetic studies

**Figure 5:**
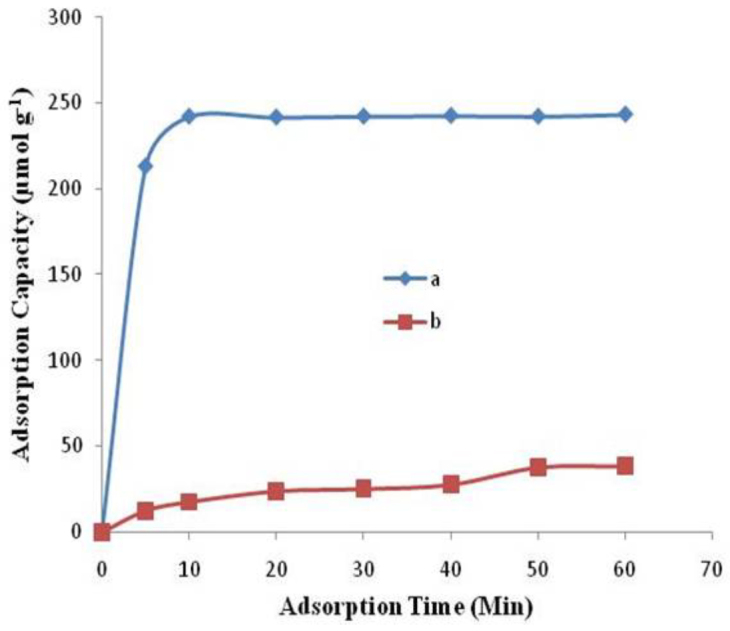
Optimization of time for maximum adsorption of Al^3+^ on (a) Al^3+^-imprinted semi- IPN cryogel composite and (b) Nonimprinted semi-IPN cryogel composite.

Figure 5 shows the adsorption capacity of both imprinted and nonimprinted semi-IPN cryogel composites for adsorption of Al^3+^ with respect to time. Initially, the adsorption of Al^3+^ ions was very fast and the maximum amount of aluminum was adsorbed within few minutes. The equilibrium was established between two phases within 10 min. Removal of Al^3+^ ions from polluted and waste water was also practiced using biosorbents derived from *Withania somnifera* [42], *Achiranthus Aspera* , and *Cassia Occidentalis* [43]. The results showed very good removal at very slow kinetics i.e. 150 and 120 min, respectively. Similarly, rice hull activated carbon (RHAC) was used to remove aluminum ions from synthetic and real wastewater, but the drawback was again the slow kinetics (i.e. 180 min) of the adsorption process [44]. Furthermore, when cleaning a real aqueous environment is desired, the kinetics of adsorption process play a major role in making adsorption material cost-effective and more applicable. The achievement of this speedy equilibrium is probably due to the shape remembrance and rapid complex formation between the Al^3+^ ions and imprinted cavities in the Al^3+^-imprinted semi-IPN cryogel composite. As it is generally known that removing a template from the imprinted polymer can leave the template-shaped cavities that correspond to the shape, size, and chemical functionality of template ion/molecule [45]. However, nonimprinted semi-IPN cryogel composite revealed relatively slow binding kinetics and lower adsorption capacity.

The mechanism of adsorption process was evaluated by fitting the experimental data into kinetic models following pseudo-first-order (PFO) and pseudo-second-order (PSO) expressions. The equations used to examine the adsorption process are given below (Eqs. 4 and 5) [46]. One of the most commonly used adsorption rate equations to describe the adsorption of a solute from a liquid phase is Lagergren rate equation which is generally known as PFO kinetic model [47]. The PFO linear equation is explained as follows:

(4)log(qe-qt)=log(qe)(k1t)/2.303

Here q_e_ is the amount of Al^3+^ sorbed at equilibrium (μmol g^-1^) and q_t_ is the amount sorbed at time t, respectively, and k_1_ denotes the rate constant of the PFO kinetic model (min_-1_).

The PSO kinetic model fits well for molecularly imprinted polymers (MIPs) due to their heterogeneous binding sites i.e. selective and nonselective binding sites. Moreover, the fast kinetics of MIPs is due to the presence of specific binding sites [48]. The linear form of the PSO kinetic model is expressed by the given relation:

(5)tqt=1kqe2+tqe

Here qe and qt have the same meaning as in Eq. (4), and k is the rate constant of PSO kinetic model (gμmol^-1^ min^-1^) .

Figure S2 presents the fitting of the PSO kinetic models for the sorption of Al^3+^ on the Al^3+^-imprinted semi-IPN cryogel composite. Table 2 shows the calculated values of the parameters corresponding to each kinetic model. It can be concluded that the value of the equilibrium adsorption capacity for Al^3+^ ions by the imprinted semi-IPN cryogel composite, calculated by PSO kinetic models, qe,calc , is very close to the experimental value,qe,exp . However, there is a huge difference between the value of qe,calc and qe,exp in case of PFO. Moreover, the correlation coefficient (R^2^) of PSO is higher than PFO. Thus, the sorption of Al^3+^ ions onto the imprinted semi-IPN cryogel composite is definitely described by the PSO kinetic model that reveals that the imprinted semi-IPN cryogel composite is heterogeneous in nature with very quick kinetics due to the high surface area and excellent affinity for aluminum. This behavior of Al^3+^ imprinted semi-IPN cryogel composite is due to successful imprinting of Al^3+^ as template.

**Table 2:** Fitting parameters of pseudo-first- and pseudo-second-order kinetic models.

	Pseudo-first-order		Pseudo-second-order	
	Al^3+^-imprinted semi-IPN cryogel composites	Nonimprinted semi-IPN cryogel composites	Al^3+^-imprinted semi-IPN cryogel composites	Nonimprinted semi-IPN cryogel composites
R^2^	0.507	0.945	0.999	0.972
K	0.014 min^-1^	0.004 min^-1^	0.016 g μmol^-1^.min^-1^	0.002 g μmol^-1^ min^-1^
Q_e_ (μmol g^-1^)	2.8	4.2	250	30.3
Q_exp._ (μmol g^-1^)	243.5	38.5	243.5	38.5

#### 3.3.3. Adsorption isotherms

**Figure 6:**
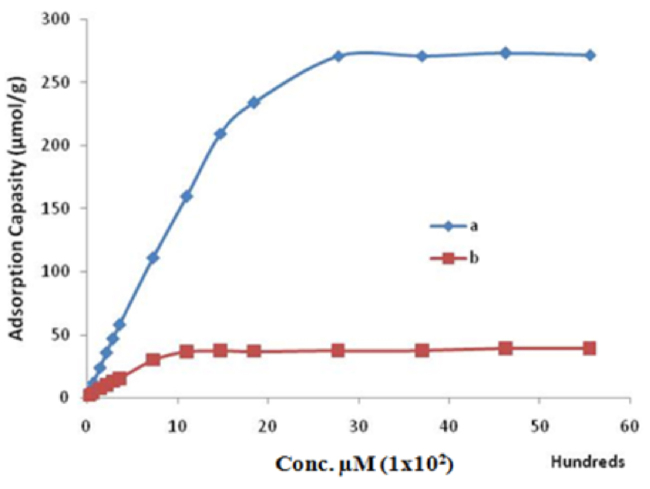
Adsorption isotherms of (a) Al^3+^-imprinted and (b) Nonimprinted semi-IPN cryogel composites for Al^3+^ ions.

Figure 6 shows the effects of initial concentration of Al^3+^ ions onto the adsorption capacity of both Al^3+^-imprinted and nonimprinted semi-IPN cryogel composites at pH 7.0. The binding isotherms of Al^3+^ imprinted and nonimprinted semi-IPN cryogel composites were determined in the concentration range of 37 to 5555 μM, the adsorption capacity was calculated using the following expression:

(6)Q=(Ci-Cf)xVm

Here Q (μmol g^-1^) expresses the mass of adsorbed Al^3+^ ions per gram of cryogel composite, C_i_ (μM) is the initial concentration of Al^3+^, C_f_ (μM) shows the final concentration, V (L) is the volume, and m (g) shows the mass of cryogel composite.

The amount of Al^3+^ions adsorbed on Al^3+^-imprinted semi-IPN cryogel composite increased with the increase in initial concentration of Al^3+^ions and reached to the highest adsorption values of 271 μmol g^-1^ . However, the maximum adsorption capacity of nonimprinted semi-IPN cryogel composite could only reach 39 μmol g^-1^ . This drastic difference in the maximum adsorption capacity of both polymers is due to the specific binding properties of Al^3+^-imprinted semi-IPN cryogel composite. The selective binding sites produced in Al^3+^imprinted semi-IPN cryogel composite are produced due to templating effect and are responsible for the efficient recognition and adsorption of Al^3+^ions in Al^3+^-imprinted semi-IPN cryogel composite. Poly (HEMA-MAGA) beads imprinted with Al^3+^ions were produced by Andaç et al. to selectively remove the Al^3+^ions from aqueous media. The highest adsorption capacity achieved by the beads was 122.9 μmol g^-1^ [49], which is much less than the adsorption capacity of Al^3+^-imprinted semi-IPN cryogel composite ( i.e. 271 μmol g^-1^) . Thus, the design of Al^3+^-imprinted semi-IPN cryogel composite also contributes to the higher adsorption capacity of prepared cryogel composite.

Ion imprinted polymers are heterogeneous materials that contain the binding regions with a broad range of selectivities and binding affinities. The binding performance of ion-imprinted polymers (IIPs) could be modeled precisely by using heterogeneous isotherm [50]. To investigate the affinity of Al^3+^-imprinted and nonimprinted semi-IPN cryogel composites, Scatchard and LF isotherms were used to analyze the adsorption data.

The Scatchard analysis was done for the obtained adsorption data of Al^3+^-imprinted and nonimprinted semi-IPN cryogel composites. It was noticed that biphasic curves with two linear lines were acquired in the case of Al^3+^-imprinted semi-IPN cryogel composite (Figure S3), which confirm the existence of two kinds of binding sites that are high- and low-affinity binding sites. However, nonimprinted semi-IPN cryogel composite did not produce a biphasic curve, which revealed that nonimprinted cryogel composite only had low-affinity binding sites.

The adsorption capacity values for Al^3+^imprinted semi-IPN cryogel composite were 292 μmol g^-1^ and 86 μmol g^-1^ for left and right biphasic slop, respectively and 54.5 μmol g^-1^ for nonimprinted semi-IPN cryogel composite. These results reveal that the higher adsorption capacity of Al^3+^-imprinted semi-IPN cryogelcomposite and presence of both high- and low-affinity binding sites is due to imprinting. Also, the value of dissociation constant KD of nonimprinted semi-IPN cryogel composite was much higher than Al^3+^-imprinted semi-IPN cryogel composite that exposes the low binding strength of nonimprinted semi-IPN cryogel composite. It also hinted that nonimprinted semi-IPN cryogel composite did not show specific adsorption. However, the specific adsorption of Al^3+^ions on Al^3+^-imprinted semi-IPN cryogel composites was achieved due to imprinting (Table 3).

**Table 3:** Fitting parameters for the Scatchard fit to the experimental adsorption isotherm of studied Al^3+^ ions on imprinted and nonimprinted semi-IPN cryogel composites.

Al^3+^-imprinted semi-IPN cryogel composite	
Linear regression equation for left slope of biphasic curve	y= –0.008x+2.42
Correlation coefficient (r) for left slope of biphasic curve	0.996
Linear regression equation for right slope of biphasic curve	y= –0.11x+8.75
Correlation coefficient (r) for right slope of biphasic curve	0.999
K_D_ (g/L) from left slope	120.5
K_D_(g/L) from right slope	9.8
Qmax (μmol/g) from left slope	292
Qmax (μmol/g) from right slope	86
Non-imprinted semi-IPN cryogel composite	
Linear regression equation	y= -0.0013x+0.07
Correlation coefficient (r)	0.992
K_D_ (g/L)	769
Qmax (μmol/g)	54.5

LF-isotherm was also used to characterize the Al^3+^-imprinted and nonimprinted semi-IPN cryogel composites (Figure S4).

The expression for LF-isotherm is given as:

(7)B=NtaFm1+aFm

Here B shows the concentration of bound and F shows the concentration of free guest in heterogeneous organization at equilibrium state. N_t_ expresses the total number of binding sites, “a” relates with the median binding affinity “K_0_ ” (K_0_ = a^1/m^) whereas “m” denotes the heterogeneity index, and these all are the fitting coefficients having physical aspects. Experimental data was used to calculate the fitting parameters of LF isotherm using MS Excel solver function by means of R^2^ value to 1 and changing N_t_, a, and m. Figure S4 in supplementary data shows nonlinear LF curves of Al^3+^-imprinted and nonimprinted semi-IPN cryogel composites. The curves were obtained by plotting B against F. It can be clearly seen that maximum bound concentration of aluminum is much higher in case of Al^3+^-imprinted semi-IPN cryogel composite as compared to nonimprinted semi-IPN cryogel composite. This vast difference in binding capacity of Al^3+^-imprinted semi-IPN cryogel composite is due to the presence of aluminum specific binding sites in Al^3+^-imprinted semi-IPN cryogel composite.

Applying the LF isotherm to experimental adsorption data of Al^3+^-imprinted semi-IPN cryogel composite is advantageous because it allows measuring the binding properties of material readily. Binding parameters of Al^3+^-imprinted semi-IPN cryogel composite could be compared directly even with the polymers having dissimilar distribution of binding sites, i.e. comparison between the binding parameters of Al^3+^-imprinted and nonimprinted semi-IPN cryogel composites. Table 4 reveals that Al^3+^-imprinted semi-IPN cryogel composite have high concentration of binding sites per gram i.e. N_t_ = 268.9 μmol g^-1^. In addition, Al^3+^-imprinted semi-IPN cryogel composite shows more heterogeneity (m = 0.08) compared to nonimprinted cryogel composite (m = 0.37), probable reason for its imprinting. The accuracy of these values can be evaluated by determining values for Ko that should fall between the limits 1/F_min_ and 1/F_max_, and by verifying the values for standard errors in the fitting coefficients are not greatly high. This study met all these requirements.

**Table 4:** Fitting parameters for the LF fit to the experimental adsorption isotherm of studied Al^3+^-imprinted and nonimprinted semi-IPN cryogel composites.

Fitting coefficients	Al^3+^-imprinted semi-IPN cryogel composite	Nonimprinted semi-IPN cryogel composite
N^t^ (μmol/g)(qm)	269 ±12	41 ±6
a (μM^-1^)	1.7 ±0.005	2.7 ±0.15
M	0.08 ±0.01	0.4 ±0.05
Ko (μM^-1^)	783	14
Limits of affinity distribution (μM^-1^)	1.53–6480	1.1–207

### 3.4. Binding specificity of Al^3+^-imprinted and nonimprinted semi-IPN cryogel composites

In order to confirm the selectivity of the Al^3+^ -imprinted and nonimprinted semi-IPN cryogel composites three different metal ions (Cu^2+^ , Ni^2+^ , and Fe^3+^) were selected for competitive adsorption. The Fe^3+^ ion was chosen as the competitor species because it has the same charge and nearly identical size and also binds well with the carboxylate groups. Ni^2+^ and Cu^2+^ were chosen because of their similar ionic radii. The adsorption capacities of Al^3+^ -imprinted and nonimprinted semi-IPN cryogel composites for Al^3+^ , Cu^2+^ , Ni^2+^ , and Fe^3+^ were recorded in Table 5.

**Table 5:** The adsorption capacity, partition coefficients, imprinting factors and selectivity coefficients of Al^3+^, Cu^2+^, Ni^2+^, and Fe^3+^ onto Al^3+^-imprinted and nonimprinted semi-IPN cryogel composites.

Analytes	Q Al^3+^-imprinted semi-IPN cryogel composite (μmol g^-1^)	Q Nonimprinted semi-IPN cryogel composite (μmol g^-1^)	K Al^3+^-imprinted semi-IPN cryogel composite (mL g^-1^)	K Nonimprinted semi-IPN cryogel composite (mL g^-1^)	IF	SC
Al^3+^	58	18	16.6	0.2	76.4	
Cu^2+^	6.9	8.2	0.36	0.2	1.5	49.6
Ni^2+^	4.3	3.4	0.2	0.08	2.3	33.9
Fe^3+^	4.8	8.3	0.2	0.2	0.9	80.3

The specificity of Al^3+^-imprinted and nonimprinted semi-IPN cryogel composites was calculated using expression for the partition coefficient, the partition coefficient K was calculated using the following relation:

(8)K=CPCs

Here C_P_ represents the amount of test analyte bound by Al^3+^-imprinted and nonimprinted semi-IPN cryogel composites, and CS is the concentration of test analyte remaining in the solution.

Moreover, selectivity of Al^3+^-imprinted and nonimprinted semi-IPN cryogel composites toward aluminum ions and related Cu^2+^, Ni^2+^ and Fe^3+^ was evaluated using imprinting factor (IF) and selectivity coefficient (SC). The following equations were used to calculate the IF and SC:

(9)Imprinting Factor(IF)=KiKc

(10)Selectivity coefficient=IFAluminum/IFi

Here K_i_ and K_c_ (Eq. 9) denote the partition coefficients of analyte for Al^3+^-imprinted and nonimprinted semi-IPN cryogel composites, respectively. IF_Aluminum_ is the imprinting factor of aluminum and IF_i_ is the imprinting factor of competitive ion in Eq. 10. The bound amount of aluminum for Al^3+^-imprinted semi-IPN cryogel composite was greater than other competitive metal ions because the template ion (Al^3+^ ion) had a relatively greater affinity for the imprinted polymer than respective metal ions. The imprinting factors obtained for Al^3+^, Ni^2+^, Cu^2+^, and Fe^3+^ using Al^3+^-imprinted and nonimprinted semi-IPN cryogel composites were found as 76.4, 2.3, 1.5, and 0.9, respectively, which correspond to the order of selectivity as Al^3+^>Ni^2+^>Cu^2+^>Fe^3+^. This may be due to the size of binding sites that are corresponding to the size of Al^3+^ ions. The ionic radii of Al^3+^, Fe^3+^, Cu^2+^, and Ni^2+^ are 67.5 pm, 73.8 pm, 87 pm, and 83 pm, respectively [49]. As the ionic radius of Al^3+^ ion is smaller than the ionic radii of competitive ions, it is obvious that other competitive ions will not be recognized by selective binding sites. The selectivity is a consequence of the ionic radius of the original metallic ion template. Nevertheless, it can also be governed by the other parameters such as the affinity between host matrix and the guest substance. A comparison of the partition coefficient (K) values for the Al^3+^-imprinted semi-IPN cryogel composite shows an increase in K for Al^3+^ while K decreases for Cu^2+^, Ni^2+^, and Fe^3+^. However, the values of K in the case of nonimprinted semi-IPN cryogel composite show minor differences due to its nonselective nature. The relative selectivity coefficient is an indicator to express metal adsorption affinity of recognition sites to the imprinted Al^3+^ ions [51,52]. These results show that the relative selectivity coefficients of Al^3+^-imprinted semi-IPN cryogel composite for Al^3+^/Cu^2+^, Al^3+^/Ni^2+^, and Al^3+^/Fe^3+^ were 49.6, 33.9, and 80.3 times greater than for the nonimprinted semi-IPN cryogel composite (Table 5).

The high value of IF achieved for aluminum reveals that imprinting technique is equally effective for semi-IPNs. Granular activated carbon and Amberlite IR-120H also offer high removal % for aluminum ions but they simultaneously remove Fe^3+^ and Mn^2+^ from industrial waste, thus do not offer good selectivity as compared to the present study [53]. Powdered modified activated carbon has also been successfully used for the removal of aluminum from some water samples but other ions (i.e. Cu^2+^, Co^2+^, Ni^2+^, or Zn^2+^) could also compete with aluminum if present in high concentrations [54].

### 3.5. Application on real water samples

**Figure 7:**
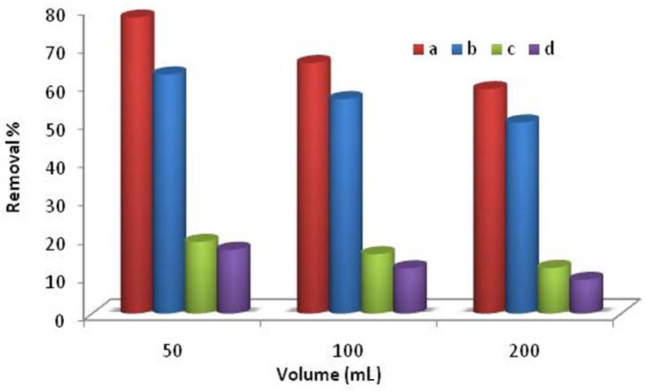
Removal of Al^3+^ from (a) Tap water and (b) River water using Al^3+^-imprinted semi- IPN cryogel composite; (c) Tap water and (d) River water by using nonimprinted semi-IPN cryogel composite.

Aluminum is identified to dissolve in water at both acidic as well as basic environment [36]. The secondary allowable limit for aluminum in potable water is set as 0.20 mg L^-1^ by Environmental Protection Agency (EPA). Thus, it is essential to remove the excess aluminum from water before being used [55]. In order to check the applicability of synthesized Al^3+^-imprinted semi-IPN cryogel composite for the removal of aluminum from real samples (tap water and river water) batch experiments were performed. Figure 7 shows the percent removal of aluminum from different volumes of tap water and river water using Al^3+^-imprinted and nonimprinted semi-IPN cryogel composite. It could be seen that maximum removal of aluminum was found at 50 mL in both tap water (77%) (Figure 7a) and river water (62%) (Figure 7b) samples using Al^3+^-imprinted semi-IPN cryogel composite. With increase in volume, percent removal decreases, which is obvious due to increase in matrix interference with increasing volume of water samples. However, the synthesized polymer is efficient and can be successfully applied for the removal of Al^3+^ ions at trace level concentrations from real water samples. On the contrary nonimprinted semi-IPN cryogel composite removed very low percent of aluminum (Figures 7c and 7d). This vast difference between percent removal of Al^3+^-imprinted and nonimprinted semi-IPN cryogel composites is due to imprinting. Imprinting makes Al^3+^-imprinted semi-IPN cryogel composite more selective and ultimately increases its adsorption capacity.

### 3.6. Desorption and reusability

**Figure 8:**
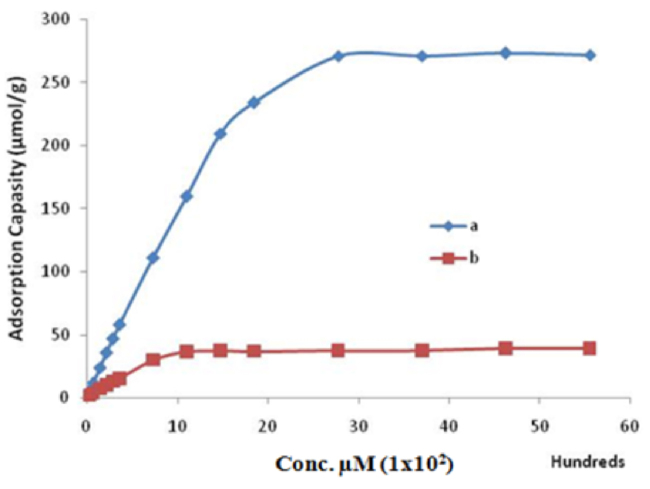
Reuse of (a) Al^3+^-imprinted and (b) Nonimprinted semi-IPN cryogel composites.

In improving water process economics, repeated use and regeneration is considered as a key factor for a commercial adsorbent. Regeneration of the Al^3+^-imprinted semi-IPN cryogel composite was confirmed by the repetition of adsorption–desorption rounds for 10 times by using the same cryogel discs. Results obtained reveal that that Al^3+^-imprinted semi-IPN cryogel composite could be used several times without noteworthy decrease in their adsorption capacity for Al^3+^ ions (Figure 8).

## 4. Conclusion

In this study, imprinted semi-IPN cryogel composite was prepared. The synthesized polymer composite showed exceedingly high performance, as marked by the fast equilibrium adsorption kinetics, very large relative selectivity coefficients, and high extraction efficiency percentages for the targeted Al^3+^ ions, even in the presence of other closely related ions. A high adsorption rate was observed at the beginning of the sorption process and saturation values were reached within 10 min at pH 7.0. The maximum Al^3+^ ions sorption capacity of the imprinted semi-IPN cryogel composite was 271 μmol g^-1^. Competitive sorption studies showed that imprinted semi-IPN cryogel composite is highly selective for Al^3+^ ions, even in the presence of Cu^2+^, Fe^3+^, and Ni^2+^ ions. The imprinted semi-IPN adsorbent could be used many times without losing adsorption capacity. Therefore, the synthesized polymer is expected to be a useful nontoxic and water-compatible adsorbent for efficient removal of aluminum from real aqueous systems at pH 7. The previously reported synthetic materials have toxicity concerns due to the use of dyes in most of them. However, cryogels are already proved as the nontoxic and biocompatible materials. They are robust, cost-effective, and they can be used effectively for longer time without deteriorating. The aluminum imprinted semi-IPN cryogel composite can easily become the part of home drinking water filtration units due to its flexibility to be in any shape without compromising tensile strength, thus providing aluminum-free drinking water at point of use. The reported composite material will open the path for imprinting of other toxic ions and organic compounds.

Supplementary Materials
