# Supplementary material for: Preparation, characterization, and binding profile of imprinted semi-IPN cryogel composite for aluminum
Source: Turk J Chem. 2020 Aug 18;44(4):901–22. doi: 10.3906/kim-2002-36 (PMC7751924; doi:10.3906/kim-2002-36)
Supplement: Supplementary file 1 — Supplementary Materials [file turkjchem-44-901-sup001.pdf]

Supplementary Information

S1. Instrumentation

Double beam UV-Visible spectrophotometer, (Agilent-Carry 100, England) equipped with the quartz cells of 1.0 cm path length had been used for the detection of Al^{3+} ions. The spectrophotometric determination of aluminum was accomplished by using chromotrop 2 R as the chelating agent. Complexation of Chromotrop 2 R with aluminum selectively forms a stable complex and gives relatively an interference free detection. Linear range of the response of the instrument had been checked by taking the absorbance of different concentrations of aluminum solution i.e. from 2 μg mL^{-1} to 150 μg mL^{-1}; all solutions were prepared through the serial dilution of stock solution of aluminum, 5mL of each solution was allowed to form complex with 50 μL of 10% solution of chromotrop 2 R. And shift in the absorbance were measured at 550 nm, the intensity of the absorbance was directly proportional to the concentration of Al^{3+}. The pH measurements of the solutions were checked by using pH-7110, pH meter, Germany.

S2. Characterization of Al^{3+}-imprinted semi-IPN cryogel composite

The surface morphology and elemental composition of the Al^{3+}-imprinted semi-IPN cryogel composite was investigated using Energy dispersive spectroscopy (EDS) and Scanning electron microscope (SEM) (JEOL, JMS-6490LV). In order to examine the surface morphology of synthesized Al^{3+}-imprinted and non-imprinted semi-IPN cryogel composites the samples were magnified at different scales. The FT-IR spectra of Al^{3+}-imprinted and non-imprinted semi-IPN cryogel composites were obtained in the range of 4000–400 cm^{-1} using a FT-IR spectrometer, Thermo Nicollet AVATAR 5700, by KBr pellet technique.

Gravimetric method has been used to determined the swelling ratios of both semi-IPN cryogel composites (imprinted and non-imprinted) in Milli-Q water. The ratio of water was described as the weight ratio of water absorbed inside swollen Al^{3+}-imprinted semi-IPN cryogel composites to dry Al^{3+}-imprinted semi-IPN cryogel composites.

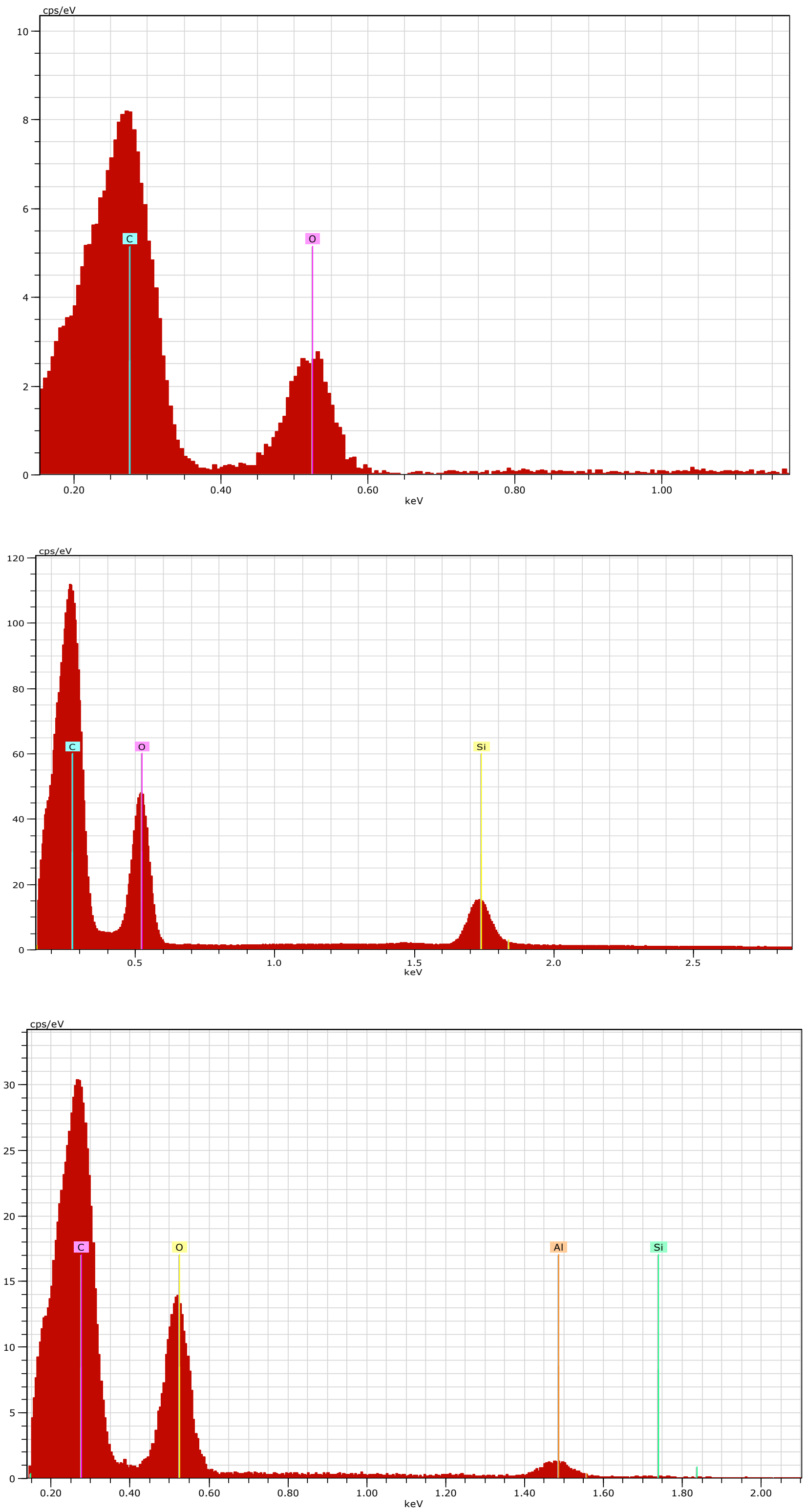

Figure S1. EDS of (a) pHEMA cryogel (b) Silanized pHEMA cryogel and (c) Al^{3+}-imprinted semi-IPN cryogel composite.

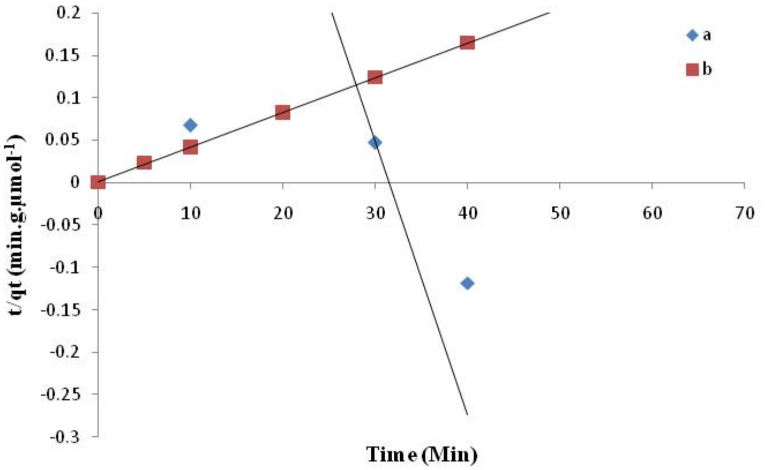

Figure S2. Uptake kinetics plots for (a) Pseudo-first order kinetic model and (b) Pseudo-second order kinetic model of Al^{3+}-imprinted semi-IPN cryogel composite for adsorption of Al^{3+}.

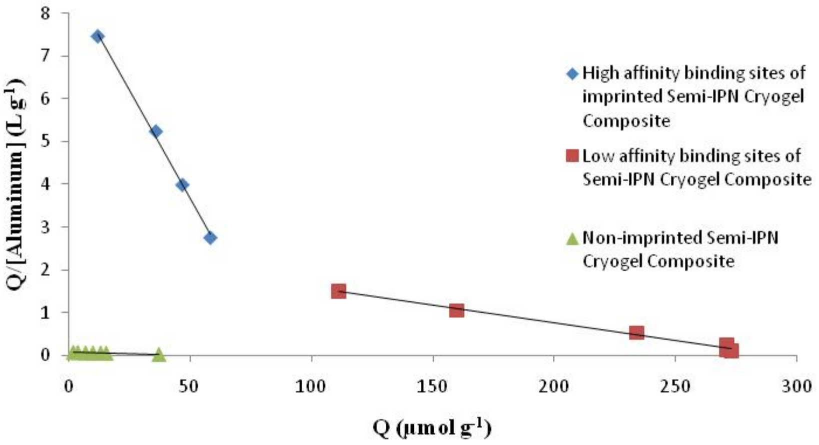

Figure S3. The Scatchard plot to estimate the binding nature of Al^{3+} imprinted and non-imprinted semi-IPN cryogel composites.

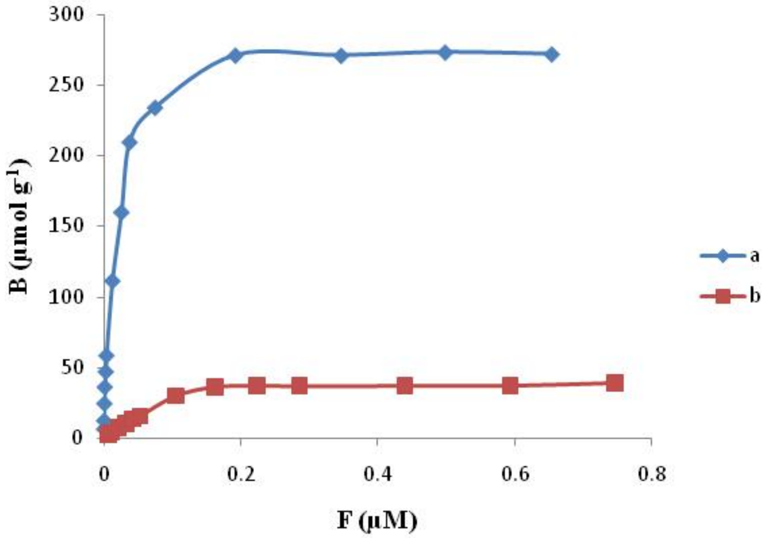

Figure S4. Binding isotherm of (a) Al^{3+}-imprinted and (b) Non-imprinted semi-IPN cryogel composite fitted to Langmuir-Freundlich (LF) isotherm.
